# Early Indications of Future Cognitive Decline: Stable versus Declining Controls

**DOI:** 10.1371/journal.pone.0074062

**Published:** 2013-09-09

**Authors:** Angela Rizk-Jackson, Philip Insel, Ronald Petersen, Paul Aisen, Clifford Jack, Michael Weiner

**Affiliations:** 1 Center for Imaging of Neurodegenerative Disease, Veterans Administration Medical Center, San Francisco, California, United States of America; 2 Mayo Alzheimer's Disease Research Center, Rochester, Minnesota, United States of America; 3 Alzheimer's Disease Cooperative Study, University of California, San Diego, La Jolla, California, United States of America; Banner Alzheimer's Institute, United States of America

## Abstract

This study aimed to identify baseline features of normal subjects that are associated with subsequent cognitive decline. Publicly available data from the Alzheimer’s Disease Neuroimaging Initiative was used to find differences in baseline clinical assessments (ADAScog, AVLT, FAQ) between cognitively healthy individuals who will suffer cognitive decline within 48 months and those who will remain stable for that period. Linear regression models indicated an individual’s conversion status was significantly associated with certain baseline neuroimaging measures, including posterior cingulate glucose metabolism. Linear Discriminant Analysis models built with baseline features derived from MRI and FDG-PET measures were capable of successfully predicting whether an individual will convert to MCI within 48 months or remain cognitively stable. The findings from this study support the idea that there exist informative differences between normal people who will later develop cognitive impairments and those who will remain cognitively stable for up to four years. Further, the feasibility of developing predictive models that can detect early states of cognitive decline in seemingly normal individuals was demonstrated.

## Introduction

Alzheimer’s disease (AD) is a debilitating illness marked by irreversible damage to brain tissue. According to recent estimates reported by the Alzheimer’s Association, as many as 5.1 million Americans suffer from AD, and if we fail to establish effective preventative measures, this number is expected to increase substantially in the coming years with our increasingly long-lived society. It is therefore of paramount importance that researchers focus efforts on better understanding the disease process in its earliest manifestation, allowing development of neuroprotective therapies that will prevent AD-related harm.

AD presents with a variety of cognitive deficits that increase in severity with disease progression including loss of memory, judgment, reasoning, and verbal fluency. While these overt symptoms can be readily assessed, the gold standard for definitive AD diagnosis remains detection of hallmark disease pathology, including plaques comprised of amyloid-beta protein (Aβ) and tangles comprised of tau protein. Alzheimer’s disease pathology exists in subjects with completely normal cognition [1]–[4], and it has been estimated that it may take at least 15 years from the time of detectable amyloid pathology until development of dementia [5]. An early stage of diminished intellectual function, called Mild Cognitive Impairment (MCI) [6]–[7], may be considered to be a prodromal stage of AD [8] especially if it can be demonstrated using biomarkers that Alzheimer’s pathology is present. Previous studies have examined MCI subject populations to determine which measures are sensitive enough to make distinctions among this population. A variety of measures have proven capable of distinguishing MCI from cognitively normal individuals, including clinical evaluation [9], [10], neuroimaging [9], [11]–[19], and biochemical [16], [19] measures. Further, clinical [9], [20], neuroimaging [21]–[25], biochemical [22] and electrophysiological [26] measures have also shown the ability to determine which MCI individuals will later suffer further decline.

While results from the MCI literature are promising, evidence suggests that disease-related neurobiological changes have already taken place prior to the onset of overt symptoms [27]–[29]. A number of studies have been aimed at identifying measures in cognitively normal subjects that are associated with future decline. Longitudinal measures of hippocampal [30]–[31] and temporal lobe [31] metabolic reductions, as well as hippocampal [32], temporal lobe [33], and overall [34] volume loss, were found to be greater for normal individuals who experienced cognitive decline relative to those who remain stable. Baseline measures within entorhinal cortex (ERC) seem to have similarly compelling associations with cognitive decline. Hypometabolism in the ERC was found to be strongly associated with the conversion from normal to MCI [31]; and baseline measures of ERC volume were found to differ significantly in a population of non-demented individuals who later converted to probable AD from those who did not convert [35]. Further, baseline pathological markers have been found to be associated with risk of future cognitive decline in cognitively normal individuals. Longitudinal examination of Aβ deposition revealed that normal healthy subjects with high Aβ at baseline are much more likely to develop MCI within 3 years relative to those with normal Aβ levels [36]. Also, baseline Aβ/tau level ratios have been shown to be predictive of whether a normal individual with a Clinical Dementia Rating (CDR) of 0 will decline to CDR>0 [37].

The Alzheimer’s Disease Neuroimaging Initiative (ADNI) provides an opportunity to further examine the issue of which changes in cognitively normal subjects predict future cognitive decline. ADNI data are collected longitudinally from over 200 normal individuals without significant cognitive complaints. Data from ADNI participants include clinical assessments of family history, cognition and behavior, MRI and PET scans, genetic information, and circulating protein levels found in the Cerebral Spinal Fluid (CSF).

The overall goal of this work is to distinguish which cognitively healthy individuals will remain cognitively stable and which will convert to MCI. We use ADNI data from normal healthy control subjects to examine whether baseline clinical, neuroimaging, or biochemical measures are associated with future conversion to MCI. Specifically, we aim to determine which clinical measures differ between decliners and non-decliners, which measures are associated with future decline, and which measures can be used to correctly classify individuals as those who will remain stable or those who will convert to MCI. Certain measures are examined a-priori, such as the individual volumes of hippocampus and ERC, cerebral glucose metabolism in the temporal lobe, and performance on logical memory tests. After testing a-priori hypotheses, a variety of additional measures are examined to determine if they could potentially identify normal subjects who will decline.

## Methods

ADNI data are publicly available through the Laboratory of Neuroimaging at UCLA (http://www.loni.ucla.edu/). Data are collected at each of over 50 ADNI sites and stored in specified ADNI data cores (see http://www.adni-info.org/Scientists/ADNIStudyProcedures.aspx) with written consent from study participants or their legally authorized representative in accordance with local IRB approvals. Use of these data was consistent with the policies set forth by the UCSF Committee on Human Research.

### 1. Study Subjects

For this analysis, we included subsets of data from all individuals that were diagnosed as normal healthy control subjects at the baseline visit, have available MRI data at baseline, and either converted to MCI at a later visit or have available data at 48 months. This subject population includes 57 individuals, 41 of which remained cognitively stable (non-converters, NC), with a CDR = 0 at all follow-up visits. The remaining 16 subjects were diagnosed with MCI (converters, CNV) at a later testing visit: 1 at 6 months post-baseline, 2 at 12 months, 5 at 24 months, 7 at 36 months, and 1 at 48 months. Some CNV subjects did not return for 48 month follow-up visits, however all NC did; therefore all CNV subjects converted within 48 months and all NC subjects remained stable for 48 months. Conversion to MCI, as outlined in the ADNI protocol, is detemined by the site physician upon clinical examination according to published criteria for MCI [7]. CNV subjects were all determined to be non-demented (CDR≤0.5, MMSE≥24), non-depressed (Geriatric Depression Scale, GDS<6).

Baseline imbalances between CNV and NC on demographic and clinical variables were assessed using the Wilcoxon Rank Sum test (α = 0.1 level). At baseline, subjects ranged in age from 63 to 90 years old with an equivalent distribution of gender among NC and CNV and no differences in education level (See Table 1).

**Table 1:** Subject population demographics.

	NC (n = 41)	CNV (n = 16)	p-value
Age (years, mean±SD)	75.93±4.59	76.58±5.27	0.32
Gender (%M)	48.8	56.2	0.77
Education (years, mean±SD)	16.49±2.71	16.06±2.91	0.55
Family history of AD (%Yes)	44.8	57.1	0.53
APOE4 carrier(%Yes, # Homozygote)	26.8, 0	43.8, 1	0.34

Demographic information describing the population of individuals who remained stable over 4 years (NC) and those who converted to MCI within that same period (CNV). Mann-Whitney tests were used to compare continuous variables and Fisher’s Exact test were used to compare categorical variables.

### 2. MRI Acquisition and Processing

Each of these subjects underwent the standardized 1.5 T MRI protocol of ADNI (see www.loni.ucla.edu/ADNI/Research/Cores/index.shtml), which included T1-weighted MRI based on a sagittal volumetric MP-RAGE sequence (TE = 4 ms, TR = 9 ms, flip angle = 8°, FOV = 256×256×166 mm). Image quality and preprocessing to correct for gradient nonlinearity and intensity non-uniformity was performed at a designated MRI Center, as described previously [38].

Images were further processed using Freesurfer software (http://surfer.nmr.mgh.harvard.edu/) to produce segmented maps with anatomical labels of 52 brain regions, yielding the volume (in mm^3^) of each region along with cortical thickness (in mm^2^) of 33 regions. The segmented maps were visually rated for accuracy by experienced quality control staff and excluded from the analysis if quality criteria were not met. A full description of the FreeSurfer processing steps can be found elsewhere [39], [40]. Finally, a subset of 5 of these regional MRI-derived volume and thickness measures were selected for primary analysis based on a-priori knowledge available in the MCI literature and included the following regions: hippocampus, entorhinal cortex, parahippocampal cortex, precuneus, and posterior cingulate cortex.

### 3. FDG-PET Acquisition and Processing

Twenty-seven of these subjects (11 CNV, 16 NC) also underwent FDG-PET imaging at baseline, and have available numerical summary data derived from the PET images. PET data were acquired on multiple instruments of varying resolution and each participating site acquired and reconstructed the FDG-PET data with the use of measured-attenuation correction and the specified reconstruction algorithm for each scanner type according to a standardized protocol (www.loni.ucla.edu/ADNI/Data/ADNI_ Data.shtml). Images were processed by the ADNI PET Core at UC Berkeley using an ROI approach [41], resulting in an average measure of glucose metabolism for 5 brain regions located in right and left angular gyri, right and left temporal gyri, and bilateral posterior cingulate gyrus.

### 4. Modeling and Analysis

To test the hypothesis that specific baseline neuroimaging measures are associated with conversion status (whether or not an individual will convert to MCI) we performed linear regression modeling. For each regression model, conversion status was used as the predictor and one of a set of different MRI-based measures were used as the outcomes. These measures included the 5 available regional FDG-PET measures (see 2.2 above) as well as the complement of FreeSurfer-derived volumetric data (see 2.1 above), which were corrected for total intracranial volume using a strategy described previously [42]. All models were adjusted for age, gender, apoE4 carrier status, and education level. P-values of hypothesis tests for effects on the 5 regional brain volumes chosen a-priori, and the 5 available regional FDG-PET measures, were considered significant at an α = 0.05 level. In a secondary exploratory analysis, all 52 regional Freesurfer-derived volumes and 33 thicknesses were considered. Regression model fits were assessed through an analysis of residuals, and p-values were corrected for multiple comparisons using the False Discovery Rate method for a-priori analyses and exploratory analyses separately.

To test the hypothesis that certain neuroimaging and cognitive measures could be used to predict an individual’s conversion status, we built a number of Linear Discriminant Analysis (LDA) models aimed at classifying individuals as CNV or NC using baseline features. LDA models built on 5 different feature sets were evaluated: 1) including the a-priori regional brain volumes derived from MRI (listed above); 2) including the MRI measures as well as a-priori selected clinical measures (ApoE4 carrier status, CSF Aβ level, and scores on delayed paragraph recall); 3) including the a-priori regional glucose metabolism measures derived from FDG-PET (listed above); 4) including the FDG-PET measures as well as a-priori selected clinical measures (same as in 2); 5) including the MRI measures and the FDG-PET measures. The models were generated using the LDA algorithm implementation available in the R (http://www.r-project.org/) MASS package (version 7.3-3). Data instances were assigned to the class for which the model-determined posterior probability was greatest.

Using a leave-one-out cross-validation procedure to evaluate the generalizable predictive capacity of these models, we generated predictions for each individual subject based on models created excluding data from that subject. The sensitivity, specificity, and overall balanced accuracy of these cross-validated models were evaluated. Those models that appeared to produce successful predictions were further assessed in two ways. First, in order to increase confidence that the results were not due to overfitting, we examined the variability in accuracies of models created using different subsets of the data. This was done by running 20 different K-fold cross-validated models (10 with K = 4 and 10 with K = 10). In K-fold cross-validation, data are split into K approximately equal groups and models are trained and tested in K folds, each fold generating predictions for data points independent of those used in model creation. Results varied depending on how the data were split, so we performed 10 random data splits for each of the K-fold cross-validated models, ensuring equivalent representations of the dataset within each fold. The mean and standard deviation of accuracies obtained from these 20 models are reported along with the accuracy of the leave-one-out model, which should theoretically be the model with least bias [43]. Second, we approximated the null distribution representing chance levels of accuracy for a given feature set. This was accomplished by randomly permuting the classification labels (eliminating any systematic information available in the data) and generating predictions from one thousand permuted leave-one-out models for each feature set. P-values for the leave-one-out model accuracies were calculated as the proportion of accuracies in the null distribution ≥ the true model accuracy for that feature set. Finally, we generated ROC curves and examined the area under the curve. Using the area under the ROC curve allows the estimate of classification accuracy to be probability-threshold free, averaging over the entire range of sensitivities and specificities [44].

All modeling and analysis statistics were performed using R statistical software (referenced above).

## Results

The CNV and NC groups displayed no differences in their demographics and a slight difference in the presence of Alzheimer’s Disease in their family history (NC, 44.8%; CNV, 57.1%) which did not approach significance. There were also no significant differences seen in CSF biomarkers of total τ (NC, 65.3±24.0; CNV, 70.5±28.1), or p-τ (NC, 22.0±7.9; CNV, 28.1±21.6); however the CNV showed moderately lower Aβ (NC, 218.3±44.4; CNV, 182.7±52, p = 0.078). The proportions of APOE4 carriers were also different, with 27% of NC and 44% of CNV carrying the ε4 allele (p = 0.341).

### 1. Baseline Group Differences in Clinical Domain

At baseline, there were no differences between CNV and NC in MMSE (assessed via proportion of subjects scoring 26, 27, 28, 29, and 30), CDR-sum of boxes (assessed via proportion of subjects scoring 0 and 0.5), delayed paragraph recall (NC, 13.2±3.1; CNV,12.1±3.5), or GDS (NC, 0.61±0.96; CNV, 0.88±1.15).

**Figure 1:**
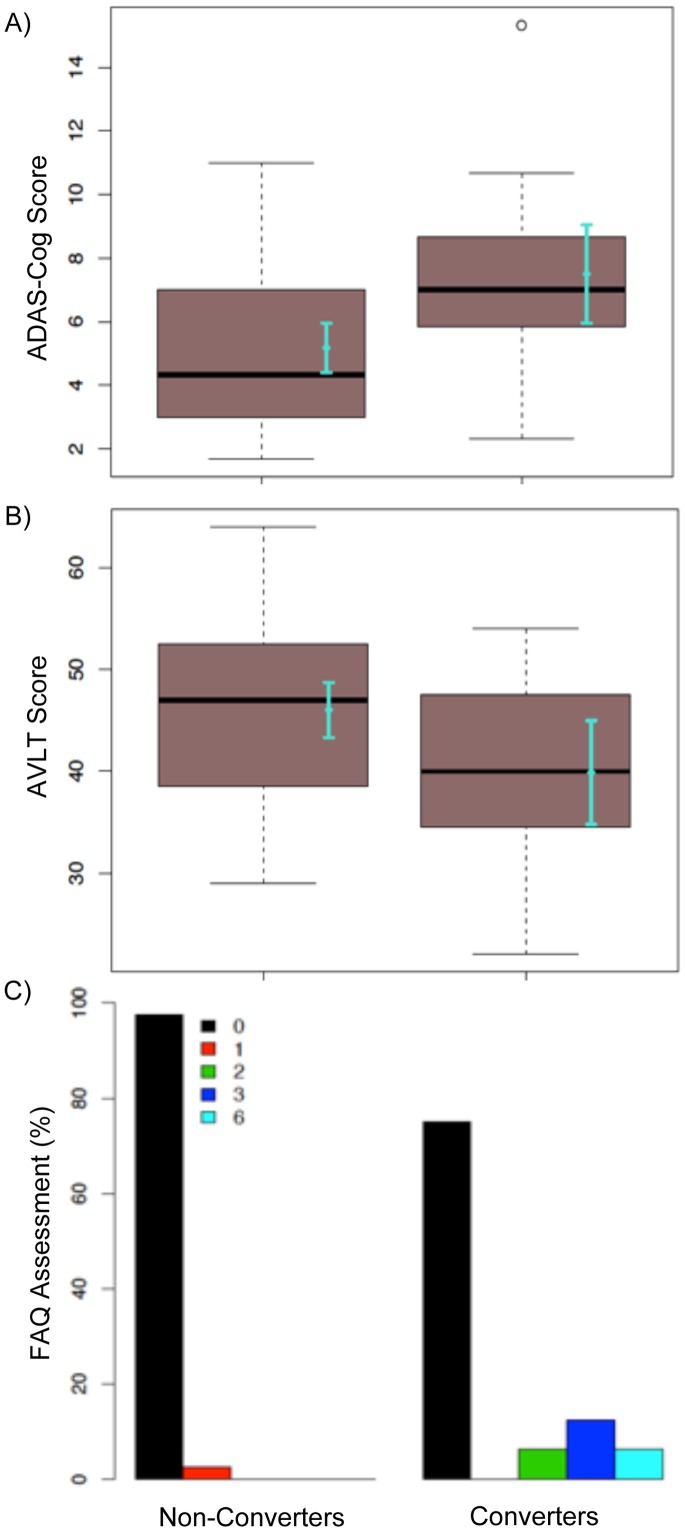
Distribution of scores on clinical assessments for converters and non-converters at baseline visit. A) Boxplot and mean with 95% CIs showing scores on the ADAS-cognitive test. B) Boxplot and mean with 95% CIs showing scores on the AVLT test. C) Bar graph showing scores on the FAQ assessment.

There were, however, significant (or near significant) differences observed between CNV and NC in a number of baseline clinical assessments including the cognitive Alzheimer’s Disease Assesment Scale – ADAS-cog (NC, 5.18±2.5; CNV, 7.5±2.9; p = 0.009), Functional Activities Questionnaire – FAQ (% of subjects [NC/CNV] with a score of: 0 [97.6/75.0], 1 [2.4/0], 2 [0/6.2], 3 [0/12.5], 6 [0/6.2]; p = 0.006), Rey Auditory Verbal Learning Test – AVLT (NC, 46.0±8.5; CNV, 39.9±9.6; p = 0.047), and delayed AVLT (NC, 8.3±3.4; CNV, 6.2±4.1; p = 0.059). See Figure 1 for associated group difference plots.

### 2. Baseline Group Differences in Neuroimaging Measures

Conversion to MCI was found to be a significant contributor to the variance observed in a number of baseline MRI-derived measures including volumes of regions such as hippocampus (p = 0.019), ERC (p = 0.040), and amygdala (p = 0.009); as well as thicknesses of pericalcarine (p = 0.014), entorhinal (p = 0.033), and insular (p = 0.036) cortical regions. Conversion status was also found to be a significant contributor to the variance observed in baseline regional FDG-PET of posterior cingulate (p = 0.007). See Tables 2 and 3 for a complete list of neuroimaging measures for which conversion status appears to show a significant (or near significant) association. Table 2 displays results from the primary analyses conducted with data from a-priori regions of interest; and Table 3 displays results from a secondary exploratory analysis in which data from each of the 52 available regional volumes and 33 regional cortical thicknesses were considered. Following correction for multiple comparisons, none of the volumetric or thickness measures reached significance and only the regional posterior cingulate FDG-PET measure remained significant (see Tables 2 & 3 for corrected p-values).

**Table 2:** Linear regression model results (a-priori measures).

Type of measure	Region	p-value
MRI-derived Volume	Hippocampus	0.019 (0.096)
	ERC	0.040 (0.100)
	PHC	0.752 (0.840)
	Precuneus	0.840 (0.840)
	PCC	0.220 (0.367)
MRI-derived Thickness	ERC	0.033 (0.132)
	PHC	0.296 (0.395)
	Precuneus	0.784 (0.784)
	PCC	0.173 (0.346)
FDG-PET regional mean	Posterior Cingulate	0.007 (0.020)
	L/R Angular	0.109 (0.152)
	L/R Temporal	0.152 (0.152)

P-values describing the significant associations of a-priori measures of interest with the conversion status variable from linear regression analyses. P-values in parentheses are corrected for multiple comparisons across all similar a-priori measures.

**Table 3:** Linear regression model results (exploratory analyses).

Type of measure	Region	p-value
Volume	Amygdala	0.009 (0.470)
	Thalamus	0.058 (0.633)
Cortical Thickness	Pericalcarine	0.014 (0.397)
	Insula	0.036 (0.397)
	Transverse Temporal	0.062 (0.437)

Selected p-values describing significant measures associated with conversion status from secondary linear regression analyses of all available measures. P-values in parentheses are corrected for multiple comparisons across all similar exploratory measures.

### 3. Predicting Conversion to MCI

LDA models created using neuroimaging measures combined with clinical measures did not achieve accuracy levels exceeding chance. However, models created with neuroimaging feature sets produced prediction accuracy levels that warranted further characterization. Using leave-one-out cross-validation, the model created using MRI-derived a-priori volumes achieved 65.3% accuracy (60.0% sensitivity, 70.6% specificity); the model created using FDG-PET measures achieved 65.5% accuracy (62.5% sensitivity, 68.4% specificity); and the model created using combined MRI & FDG-PET measures achieved 81.2% accuracy (80.0% sensitivity, 82.4% specificity).

**Figure 2:**
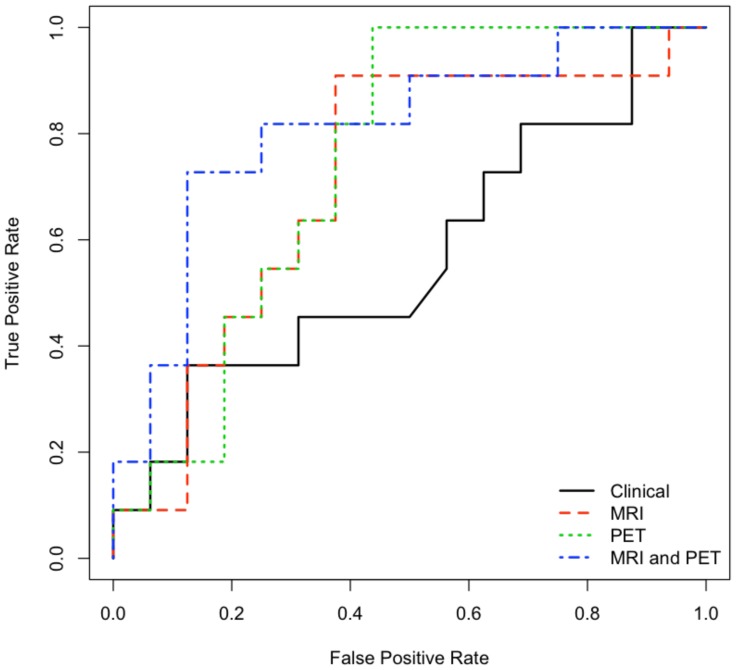
ROC curve displaying performance of predictive models built using subsets of data including clinical measures, MRI-derived features, PET-derived features, and MRI-PET combined feature sets.

The K-fold cross-validation approach revealed that the MRI-only and PET-only models varied by less than 5 percentage points and averaged accuracy levels similar to those seen in their corresponding leave-one-out models (mean±SD: 66.0±3.9% and 63.2±4.2% respectively). However, the MRI-PET model displayed much more variability and averaged an accuracy level nearly 10 percentage points lower than its leave-one-out counterpart (mean±SD: 73.8±7.3%). Further examination of model sensitivity and specificity via ROC analysis is displayed in Figure 2.

The permutation analysis generated p-values for each model set, demonstrating that the MRI-only and PET-only models did not differ significantly from chance accuracy (*p* = 0.122 and *p* = 0.096 respectively). However, the MRI-PET model maintained significantly above chance accuracy for leave-one-out (*p* = 0.005) as well as each of the K-fold model sets (*p*<0.05).

## Discussion

The major findings from this work are: 1) Significant differences in baseline clinical assessments (ADAS-cog, FAQ, and AVLT) exist between normal, cognitively healthy individuals who will suffer cognitive decline within 48 months and those who will remain stable for that period. 2) Significant associations exist between certain baseline neuroimaging measures (hippocampal and entorhinal volume as well as posterior cingulate metabolism) and an individual’s future cognitive state (specifically, whether or not the cognitively healthy individual will suffer cognitive decline within 48 months or remain stable for that period). 3) Simple LDA models built with baseline features derived from MRI and FDG-PET measures are capable of successfully predicting whether an individual will convert to MCI within 48 months or remain cognitively stable. Taken together these findings suggest that individuals who suffer from cognitive decline express subtle, but informative, differences from stable individuals up to 4 years prior to displaying overt neuropsychological symptoms of decline.

The first major finding was that clinical assessments, including the ADAS-cog, AVLT, and FAQ, showed group differences between CNV and NC. The ADAS-cog evaluates the domains of memory, reasoning, language, orientation, attention and praxis. Scored in terms of errors, the higher scores obtained by CNV at baseline reflect poorer performance as compared to NC. ADAS-cog has been shown to be particularly informative in classifying normal controls from individuals with MCI or AD, and also in predicting MCI to AD conversion [20]. The AVLT is a list-learning task that is designed to assess multiple cognitive parameters associated with learning and memory. Higher scores on AVLT by NC as compared to CNV reflect greater list memory in this group, and near significant differences in delayed AVLT suggest that NC may also display more robust memory retention than CNV. Chen et al. have previously found significant group differences between stable MCI and MCI to AD converters in both ADAS-cog and delayed AVLT [11], thus demonstrating the sensitivity of these tests to alterations in cognition associated with increased risk of developing dementia. Results from the present study further extend these findings to detection of subtle impairments associated with increased risk of cognitive decline from normal cognitive health to states of MCI.

FAQ is based on a partner or caregiver’s assessment of an individual’s ability to carry out complex activities of daily living. Scoring for each functional domain indicates a disability in performance, thus the greater proportion of CNV scoring >0 on these domains signifies increased impairment in activities of daily living. Impairment in activities of daily living has been shown to be already present in individuals with MCI [10], however the present findings indicate that these impairments may manifest even prior to the onset of clinical decline and could be considered, along with the ADAS-cog and AVLT, amongst the earliest clinically assessed indicators of risk for future cognitive decline in normal populations.

Differences were observed between MCI converters and cognitively stable individuals in their familial history of AD occurrence as well as the proportions of APOE4 carriers, with a higher incidence of familial AD and higher proportion of E4 carriers being seen in MCI converters. While these differences were not statistically significant in this small sample of data, likely due to insufficient power, they do suggest a potential connection between known genetic risks for developing AD and the risk for cognitively healthy individuals to develop cognitive impairments.

Our second major finding was that neuroimaging measures were significantly associated with an individual’s conversion status (i.e. whether or not they would convert to MCI within 4 years), These include both MRI-derived structural measures as well as FDG-PET-derived metabolic measures. In contrast to the genetic factors, which convey only static information about risk, the neuroimaging measures appear more sensitive to the risk of near-term cognitive decline (within 48 months). This is perhaps due to the fact that neuroimaging measures are dynamic, and can therefore reflect processes underway as an individual nears the point of cognitive decline.

Conversion from a normal control to MCI was most notably associated with functional measures of average glucose metabolism (MRglc) within the posterior cingulate region in the present study. This aligns well with previous findings of hypometabolism within this region associated with early stages of MCI [12], [17], [45]–[48]. Hypometabolism in the hippocampal and entorhinal cortices have also been found to be associated with cognitive decline. Researchers have found that longitudinal measures of hippocampal [30], [31] and temporal neocortical [31] MRglc reductions were greater for normal individuals who experienced cognitive decline relative to those who remain stable and concluded that, in the normal stages of cognition, these longitudinal measures of the rate of hypometabolism are sensitive markers of future cognitive decline. Further, baseline measures of hypometabolism in the ERC were found to be strongly associated with the conversion from normal to MCI [31]. We did not have specific measures of hippocampal or entorhinal hypometabolism in the current study, as we were working with numerical summary data averaged over much broader brain regions. It is possible that future efforts to create more spatially refined summary measures of cortical hypometabolism, including smaller regions such as hippocampus, could produce findings equivalent to those presented here relating to the posterior cingulate. However, it can be challenging to obtain consistent and reproducible measures of these smaller regions due to the variability of brain structure as well as protocols for delineation of regional borders.

Conversion status was also associated with structural measures of brain volume in a-priori selected regions of hippocampus and ERC, as well as ERC thickness. This is also in line with several previous reports demonstrating atrophy in these temporal regions early in the disease process [49]–[52]. Longitudinal, but not baseline, measures of volume loss in the hippocampus [32] and medial temporal lobe [33] were found to be greater in normal patients who converted to MCI than in those who remained cognitively stable. However, baseline measures of ERC were found to differ significantly in a population of non-demented individuals who reported memory complaints and would later convert to probable AD from those who did not convert [35]. Further, a 2009 study of structural MRI biomarkers of early AD found that hippocampal volume and ERC thickness show a pattern of progressive atrophy from normal control individuals to those with single-domain MCI, to those with multi-domain MCI, to those with early AD [53]. Taken together, this work suggests that these markers are sensitive to progressive risk of cognitive decline from earlier to later phases of disease.

Our third major finding is that statistical models using feature sets of baseline neuroimaging measures are capable of successfully predicting which cognitively normal individuals will convert to MCI and which will remain stable. It is important to contrast the true predictive models presented here with previous work whose model outcomes suggest the predictive capacity of certain measures, but do not directly test them. For example, researchers have been able to use basic associative models to predict which normal individuals will convert to MCI using MRglc reductions in ERC [31] or using the rate of atrophy in the medial temporal lobe [33]; and also use hippocampal volume to predict which non-demented individuals would develop dementia [54]. In these studies, the models were assessed using the same data that was used to create them, and therefore the ‘predictions’ are not generalizable. True assessment of predictive models requires some form of validation in which the data used to make predictions was not used in the creation of the predictive model. Recent efforts have focused on producing true predictive models that are capable of predicting an individual’s risk for disease progression. These models have demonstrated the ability to predict which MCI individuals will convert to AD using cortical thickness measures [55], maps of regional grey matter distribution [56], and multimodal biomarkers derived from combined MRI and FDG-PET [24] and MRI/FDG-PET combined with CSF biomarkers [19]; while others have used neurophysiological measurements to predict which MCI individuals will progress with further cognitive decline [26]. In the current study, we extend this predictive approach into the realm of pre-decline cognitively normal individuals. Using a-priori selected features derived from neuroimaging measures, we were able to successfully predict which normal individuals would convert to MCI within 4 years and which would remain cognitively stable.

In this particular instance, neither the structural features derived from MRI nor the functional measures of glucose metabolism derived from FDG-PET alone were sufficient to produce a successful predictive model beyond the accepted level of significance; but models created using multimodal feature sets from both MRI and FDG-PET were capable of predicting conversion to MCI with up to 81% accuracy, significantly exceeding chance accuracy levels. However, it is quite possible that MRI or PET measures alone could produce significantly successful predictive models, as our study is limited by a small sample size. In fact, in most cases, the MRI-only and PET-only models predicted MCI conversion with above-chance accuracy levels (65–70%), and this performance approached significance but simply failed to exceed the accepted *p*<0.05 cutoff. Further examination of model sensitivity and specificity via ROC analysis also demonstrates similar behavior of all neuroimaging-based models, with clear superiority over models based on selected clinical measures.

While the predictive models presented in the current study were cross-validated to examine generalizability, replication of the results of these models in a different sample of subjects would further demonstrate their validity and robustness. The second phase of ADNI, currently underway, provides a new sample of controls, each with all of the available measures used in the present study, as well as amyloid PET scanning and structural MRI at 3T.

As discussed above, results from the current study demonstrate that characteristics of hippocampal and entorhinal cortical structure are sensitive enough to express subtle differences between normal individuals who will remain cognitively stable from those who will later develop MCI. In the context of the associated literature, present results support the notion that these measures could provide powerful biomarkers representing either the very early stages of cognitive decline or the risk for developing future cognitive impairments. Our secondary analysis also points to a number of additional regional measures of brain structure that appear to be associated with a healthy individual’s risk of developing MCI. Amygdalar volume is one such measure. Structural measures of the amygdala have previously shown potential for differentiating groups in early phase cognitive decline [14] as well as a strong association with the risk to develop AD dementia [54]. In relation to the present study, it is important to keep in mind that the tests performed in the secondary analysis were purely exploratory and statistical significance of these effects did not survive correction for multiple comparisons.

It is apparent from the literature that a variety of measures, from clinical assessments to direct observations of brain structure and function, could potentially provide informative markers reflecting early phase cognitive decline. In fact, many of the measures identified in the present study have demonstrated associations, such as significant correlations between hippocampal volume, ADAS-cog and AVLT [11]. While some have suggested a specific sequence of alterations in these markers relation to the course of AD [57], longitudinal studies are needed to discern the temporal and causal relationships of these measures. Further, longitudinal analyses could provide even more sensitive markers of disease states, as studies have suggested that the rates of change of these measures may prove much more informative than their values at baseline [30]–[33].

We have chosen to use the clinical diagnosis of MCI as our standard for cognitive decline in normal individuals, but it is important to keep in mind that individuals with MCI belong to a heterogeneous group that may include people who progress to AD and those who may be suffering from other conditions of cognitive decline [58]. Therefore, while much of what is described fits very well with previous AD-specific findings, the results presented here may not be reflective of AD-specific degenerative processes and may instead reflect more general processes associated with early stages of cognitive decline. In any case, the findings from this study support the idea that there exist subtle, but informative, differences between normal people who will later develop cognitive impairments and those who will remain cognitively stable for up to four years. Further, we have demonstrated the feasibility of developing predictive models that can detect early states of cognitive decline in seemingly normal individuals. Such models would be of particular value for the development of preventative treatments, providing quantifiable metrics of projected decline-related alterations.
